# TNFα-CXCR1/2 partners in crime in insulin resistance conditions

**DOI:** 10.1038/s41420-024-02227-5

**Published:** 2024-12-03

**Authors:** Vanessa Castelli, Housem Kacem, Laura Brandolini, Cristina Giorgio, Marta Sofia Scenna, Marcello Allegretti, Annamaria Cimini, Michele d’Angelo

**Affiliations:** 1 Department of Life, Health and Environmental Sciences, University of L’Aquila, L’Aquila, Italy; 2 Dompé Farmaceutici Spa, via Campo di Pile, 1, L’Aquila, Italy; 3 Dompé Farmaceutici Spa, via T. De Amicis, 95, Naples, Italy; 4 Sbarro Institute for Cancer Research and Molecular Medicine, Temple University, Philadelphia, USA

**Keywords:** Pharmaceutics, Target validation

## Abstract

Type 2 diabetes mellitus (T2D) is defined by chronic hyperglycemia due to insufficient insulin secretion or activity and decreased insulin sensitivity, known as insulin resistance (IR). This condition leads to oxidative stress and inflammation, increasing the risk of systemic inflammatory diseases. Obesity and a sedentary lifestyle are major risk factors for IR and T2D. Various metabolites act as mediators of IR by disrupting communication between organs. Lipids, including free fatty acids and short-chain fatty acids, along with intracellular lipotoxins, impair insulin function and mitochondrial activity, contributing to IR through direct and indirect mechanisms such as oxidative stress and inflammation. Our research explores the role of TNFα and CXCR1/2 in IR conditions, emphasizing their interactions and potential as therapeutic targets. In this study we selected two models of IR, adipocytes and hepatocytes, since are key players in glucose and lipid metabolism. To develop IR model, TNFα was used as challenge and we focused on investigating the role of CXCR1/2 inhibition. We assessed glucose uptake, insulin signaling pathways, and gene expression related to IR. Cells treated with TNFα showed reduced p-Akt and increased p-JNK levels, indicative of IR. In contrast, CXCR1/2 inhibition restored p-Akt levels and reduced p-JNK levels, suggesting improvements in insulin signaling and glucose uptake. Furthermore, CXCR1/2 inhibition counteracted the TNFα-induced decrease in IGF expression and restored GLUT2 expression, indicating enhanced insulin sensitivity. These results underscore the pivotal role of CXCR1/2 in modulating the inflammatory response and insulin signaling in IR conditions in both IR models. CXCR1/2 inhibition can mitigate IR and improve glucose metabolism. Thus, targeting the TNFα-CXCR1/2 pathway presents a promising therapeutic approach for managing IR and T2D. Further investigation is necessary to understand the clinical implications of these findings and develop effective treatments for patients with IR and T2D.

## Introduction

Type 2 diabetes mellitus (T2D) is a metabolic condition depicted by chronic hyperglycemia, inadequate insulin secretion or activity, and impaired insulin sensitivity, also known as insulin resistance (IR) [1, 2]. Long-term hyperglycemia leads to increased oxidative stress and inflammation, leading to a higher risk of chronic inflammatory systemic diseases and related problems. Obesity, visceral adiposity and sedentary lifestyle are common risk factors for IR and T2D. Accumulating evidence suggests that in T2D condition, interorgan communication is disrupted by a variety of metabolites acting as mediators, contributing significantly to IR. Various metabolic signals circulating in the body, including specific lipids, amino acids, and ketoacids, have been identified as initiators or contributors to IR, as well as mediators of decreased insulin sensitivity between different organs [3]. Lipids like free fatty acids, acetate, and palmitoleate from adipose tissue, along with short-chain fatty acids from the gut, can influence the liver and skeletal muscle. Intracellular lipids including diacylglycerols and sphingolipids can act as lipotoxins, preventing insulin action in myocytes and hepatocytes. In states of IR, metabolic flexibility is often compromised, indicating abnormal mitochondrial function characterized by alteration in mitochondrial respiratory activity, dynamics, and turnover. Lipid-induced IR is probably due to both direct actions of lipotoxins on insulin signaling and indirect effects stemming from compromised lipid metabolism and mitochondrial functionality, which generate signals like oxidative stress and inflammation, further inhibiting hepatic insulin activity [4–8].

In addition to the liver, white adipose tissue plays a crucial role in maintaining systemic energy balance. While each organ has its specialized functions, they must collaborate to regulate metabolism throughout the entire body. Both adipose tissue and the liver can adapt to excess energy by storing triglycerides up to a certain point without significant disruption to metabolism. However, when WAT surpasses its threshold for storing fat, it becomes dysfunctional, leading to metabolic inflexibility, inflammation, and abnormal secretion of adipokines. This failure in lipid storage and mobilization by adipose tissue results in an overflow of lipids throughout the body, particularly in the liver.

It is well established that TNF-α plays a central role in disrupting insulin signaling in various tissues, including adipocytes and hepatocytes, by promoting pro-inflammatory pathways such as NF-κB and JNK. These pathways inhibit key components of the insulin signaling cascade, leading to reduced glucose uptake and insulin sensitivity in both liver and adipose tissues [9].

Indeed, TNF-α can interfere with insulin signaling by altering crucial points in the insulin pathway in different tissues, i.e., the liver, skeletal muscle, and adipose tissue. TNF-α can disrupt the normal function of the insulin receptor and its downstream molecules, including insulin receptor substrate 1 (IRS1) and Akt substrate 160 (AS160). These molecules are involved in the translocation of glucose transporter 4 (GLUT4) to the plasma membrane, which allows glucose uptake into the cells.

Lipotoxins, such as free fatty acids (FFAs), play a crucial role in inducing insulin resistance through the activation of pro-inflammatory pathways. These lipids can directly stimulate the production of TNF-α in adipose tissue and liver, leading to local inflammation and interference with insulin signaling. Specifically, TNF-α impairs insulin receptor signaling by promoting serine phosphorylation of insulin receptor substrate-1 (IRS-1), which leads to decreased insulin sensitivity. Studies have shown that circulating lipotoxins can trigger this process by activating toll-like receptors (TLRs), which in turn promote the expression of pro-inflammatory cytokines like TNF-α. This mechanism is particularly significant in the context of obesity and metabolic syndrome, where excess lipid accumulation exacerbates inflammation [10]. As Borst et al. describe, the interaction between lipids and TNF-α is critical in understanding the molecular basis of insulin resistance [11].

TNF-α can also induce the expression of various proteins that can inhibit insulin action, such as protein tyrosine phosphatase 1B (PTP1B), fetuin-A, fibroblast growth factor 21 (FGF21), and retinol-binding protein 4 (RBP4) [12].

Moreover, in T2D, the pro-inflammatory action of the cytokines involves the NF-kB and JNK pathways. The pharmacological treatments against these players resulted in the modulation of insulin sensitivity in different animal species. In humans, it has been observed that the improvement of IR described with the antidiabetic thiazolidinediones or statins is mainly ascribed to anti-inflammatory activity.

It has been described that inflammation-dependent pathways have a crucial pathological role in the IR condition in adipocytes and in hepatocytes [13].

However, the specific contribution of the TNFα-CXCR1/2 axis to insulin resistance remains underexplored. While TNF-α’s general role in inflammation is well documented, the interplay between CXCR1/2 signaling and TNF-α in modulating insulin sensitivity, particularly in metabolic tissues like adipocytes and hepatocytes, is not fully understood [14]. Our study addresses this gap by investigating whether the inhibition of CXCR1/2 could restore insulin signaling disrupted by TNF-α.

In this scenario, the CXCR1/2 pathway is emerging as implicated in several phases of diabetes development and progression. In T2D, adipose tissue inflammation is believed to exert a significant role, with CXCL8 proposed as a contributing factor. In T2D, chronic low-grade inflammation within adipose tissue contributes significantly to insulin resistance and disease pathogenesis [15]. CXCL8, also known as interleukin-8, is a chemokine involved in the recruitment and activation of immune cells during inflammation. Elevated levels of CXCL8 have been observed in obesity and T2D, suggesting its involvement in adipose tissue inflammation and insulin resistance [16]. Moreover, recent studies have highlighted the importance of chemokine receptors in non-inflammatory tissues, expanding our understanding of their roles beyond immune cell trafficking. CXCR2 receptors, for instance, expressed in smooth muscle cells, have been involved in the onset of IR [17]. This highlights a novel aspect of chemokine signaling in metabolic regulation. Furthermore, the expression of CXCR1/2 in human adipocytes has been reported, underscoring their significance in regulating insulin sensitivity [16] and metabolic homeostasis [18].

Indeed, we demonstrated in a previous work the positive effect of the inhibition of CXCR1/2 in 3T3-L1 adipocytes upon inflammatory and diabetic conditions [18]. Specifically, we found that inhibiting CXCR1/2 through Ladarixin (a small molecule that acts as a selective dual antagonist of CXCR1 and CXCR2) improved the insulin sensitivity of 3T3-L1 adipocytes by dampening inflammation and amelioration insulin signaling. These results were also demonstrated in human adipocytes derived from subcutaneous fat tissue of obese patients with T2D. Indeed, ladarixin reduced the size and number of adipocytes, decreased the expression of pro-inflammatory cytokines, and increased the expression of GLUT4 and PPARγ [18].

Therefore, we hypothesized that the activation of CXCR1/2 may have a detrimental role in the insulin sensitivity, thus inducing resistance and its inhibition may have positive effects on improving insulin sensitivity and preventing or treating metabolic disorders such as diabetes mellitus.

The interplay between the CXCR1/2 pathway and TNF-α is pivotal in understanding the mechanisms underlying IR and inflammation in diabetes. TNF-α by binding to its receptors, particularly TNFR1 and TNFR2, leads to the activation of downstream signaling cascades, such as the nuclear factor kappa B (NF-κB) pathway [19]. Activation of NF-κB induces the expression of various pro-inflammatory genes, involving chemokines, cytokines, and adhesion molecules, thereby amplifying the inflammatory response [20]. Interestingly, the CXCR1/2 pathway has been shown to interact with TNF-α signaling, thereby modulating the inflammatory response and insulin sensitivity. Activation of CXCR1/2 receptors by their ligands, including IL-8, has been shown to enhance TNF-α-mediated inflammatory responses [21]. Conversely, inhibition of CXCR1/2 signaling attenuates TNF-α-induced inflammation, suggesting a regulatory role for CXCR1/2 in modulating TNF-α signaling [22]. Thus, the interaction between CXCR1/2 and TNF-α signaling pathways may contribute to the dysregulation of insulin sensitivity and the pathogenesis of diabetes.

Further elucidation of these interactions may provide insights into new therapeutic strategies for the treatment of diabetes and related metabolic disorders. By investigating the impact of CXCR1/2 inhibition on TNF-α-induced IR, this study seeks to provide insights into the potential therapeutic targeting of chemokine receptors to improve insulin sensitivity.

Building upon these findings, the present study aims to additional elucidate the role of CXCR1/2 signaling in IR using TNF-a induced insulin resistance in vitro models [23].

For these reasons, we focused on the effects of the inhibition of CXCR1 and/ CXCR2 in TNFα-induced IR in adipocytes and hepatocytes. In this study, we selected two models of IR, adipocytes and hepatocytes, since are key players in glucose and lipid metabolism. Specifically, adipocytes regulate systemic energy balance and lipid storage, while hepatocytes control glucose homeostasis. These cells are critically involved in the pathophysiology of type 2 diabetes and insulin sensitivity, making them ideal models for studying the inflammatory mechanisms of IR. To develop IR model, TNFα was used as challenge and we focused on investigating the role of CXCR1/2 inhibition. To this purpose, the receptors were either silenced by siRNA or by the use of a dual inhibitor of CXCR1 and CXCR2 in both cellular models. The data obtained point toward a key role of CXCR1/2 signaling in TNF-α dependent IR conditions.

## Results

### Evaluation of the involvement of CXCR1/CXCR2 in IR-adipocytes and hepatocytes

IR-adipocyte and -hepatocyte models were developed using TNF-α challenging to mimic the autocrine role of TNF-α on adipose tissue [24] and liver [11]. The establishment of IR condition was confirmed by glucose uptake assay. The role of CXCR1/2 in IR was dissected by silencing both CXCR1/CXCR2 expressions (through siRNA) or by inhibiting CXCR1/2 with a dual allosteric inhibitor (Ladarixin) or with a neutralizing antibody. The effective silencing of these receptors was confirmed by Real-Time PCR in both cell lines; mRNA expression of CXCR1 was found reduced compared to both control (siRNA CXCR1 vs Scramble) and IR conditions (siRNA CXCR1 + TNF-a vs Scramble + TNF-a). Similar findings were observed for CXCR2 (suppl. Fig. 1).

**Fig. 1:**
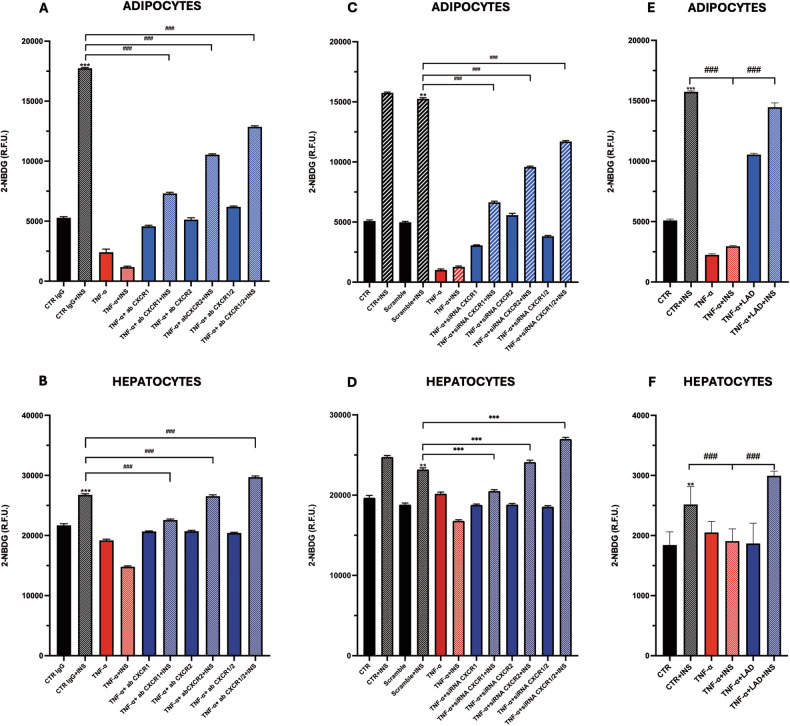
Glucose uptake evalution in adipocytes and hepatocytes. Glucose Uptake of silenced adipocytes (**A**), of silenced hepatocytes (**B**), of neutralized adipocytes (**C**) and neutralized hepatocytes (**D**) are shown. Glucose Uptake of treated adipocytes (**E**) and hepatocytes (**F**) with Ladarixin (LAD) are shown. Data are mean ± SE of 3 different experiments; One way ANOVA; ^#^*p* < 0.05, ^##^*p* < 0.005^, ###^*p* < 0.0005 vs the silencing/inhibition/treated condition (*N* = 3), *p* < 0.05, **p < 0.005, ***p < 0.0005 vs. their respective condition without INS stimulation (*N* = 3).

Glucose uptake was evaluated under all inhibitory conditions described and in both models (Fig. 1A–F). It is possible to appreciate that the inhibition by Ladarixin showed a stronger effect on glucose uptake compared to gene silencing (due to the incomplete silencing of the receptors by siRNA) but also to neutralization. Interestingly, in adipocytes, CXCR2 siRNA induced a stronger uptake than the silencing of CXCR1, thus suggesting a possible pivotal role in this tissue of CXCR2 in IR (Fig. 1A, C, E).

Once the role of CXCR1/2 has been confirmed through gene silencing or dual receptors inhibition or neutralization, the subsequent experiments were conducted using the dual allosteric inhibitor Ladarixin to confirm its efficacy and reproducibility. We first focused on adipocytes and the evaluation of tissue-specific markers involved in IR conditions and then on IR hepatocytes.

### Adipokines secretion and metabolism in adipocytes

**Fig. 2:**
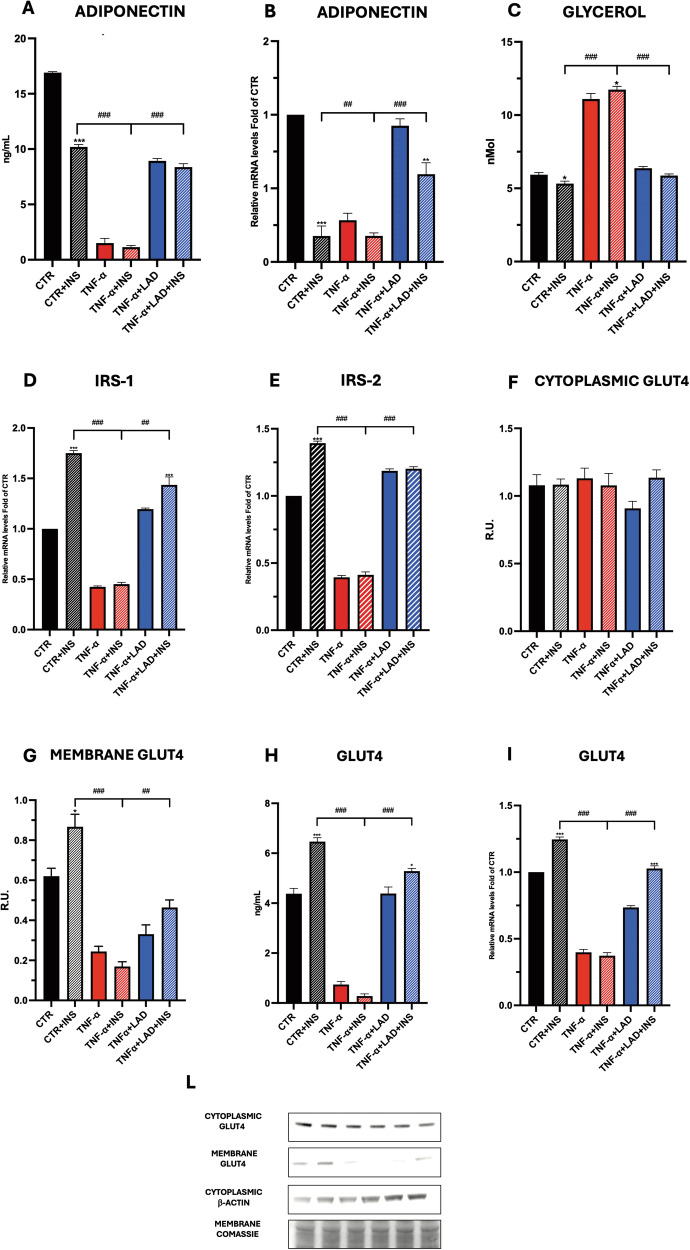
Metabolism in adipocytes. **A** Adiponectin ELISA assay, **B** Adiponectin Real-Time PCR, **C** Glycerol, **D** IRS1 and **E** IRS2 expressions by Real-Time PCR in adipocytes upon different conditions. **F**, **G**, **L** Western blotting of cytoplasmic fraction and of the membrane and GLUT-4 and relative densitometric analyses. A representative figure is shown. **H** ELISA assay for the quantification of GLUT-4 protein level, **I** GLUT-4 expression by Real-Time PCR in adipocytes are reported. Data are mean ± SE of 3 different experiments; One way ANOVA. ^#^*p* < 0.05, ^##^*p* < 0.005, ^###^*p* < 0.0005 vs. the other conditions with the same INS stimulation (*N* = 3); *p < 0.05, **p < 0.005, ***p < 0.0005 vs. their respective condition without INS stimulation (*N* = 3).

Adiponectin secretion was found to decrease in IR conditions (TNF-a and TNF-a+INS, 1.5 ± 0.4 and 1.1 ± 0.1, respectively) with respect to the control (CTR + INS, 10.1 ± 0.2 ng/ml) (Fig. 2A). Notably, inhibition of CXCR1/2 by Ladarixin improved the hormone secretion (TNF-a+LAD, TNF-a+LAD + INS; 8.9 ± 0.2, and 8.3 ± 0.3 ng/ml, respectively). These results were further confirmed by Real-Time PCR for adiponectin expression (Fig. 2B).

Glycerol concentration in the IR conditions (TNF-α and TNF-α + INS, 11.0 ± 0.3, 11.7 ± 0.2 nmol, respectively) was approximately two-fold higher than in control conditions (5.9 ± 0.1 nmol), while CXCR1/2 inhibition significantly reduced its level (TNF-a+LAD, TNF-a+LAD + INS; 6.3 ± 0.1, and 5.9 ± 0.1 nmol, respectively Fig. 2C). Adipocytes secrete leptin in direct proportion to adipose tissue mass [25].

### Adipocytes: Analysis of the pathways involved in IR

GLUT4 is the insulin-regulated glucose transporter found primarily in adipose tissues. Increased insulin levels trigger glucose uptake into the cells. GLUT4 is stored in the cell in transport vesicles and is promptly incorporated into the plasma membrane of the cell when insulin binds to membrane receptors. In our adipocyte model, GLUT4 levels were found to decrease in IR conditions (TNF-a and TNF-a +INS, 0.7 ± 0.1 and 0.3 ± 0.1 ng/ml) with respect to the control (CTR, CTR + INS, 4.3 ± 0.2 and 6.5 ± 0.1 ng/ml, respectively) and increased upon CXCR1/2 inhibition (TNF-a+LAD, TNF-a+LAD + INS 4.4 ± 0.2 and 5.3 ± 0.1 ng/ml, respectively) (Fig. 2H). These results were also confirmed by gene expression and protein analysis (Fig. F, G, I, L). Specifically, data obtained in a series of experiments showed that using a total protein extract for the experiment, the levels of this protein did not significantly vary among conditions (data not shown). Therefore, to better evaluate the levels of the membrane transporter GLUT4, we performed western blotting from a membrane protein extract (Fig. 2G). The results show how IR conditions (TNF-a and TNF-a+INS) decreased GLUT4 transporter at membrane levels, while CXCR1/2 antagonism increased the protein at membrane level, thus suggesting a role of these receptors in glucose metabolism.

IRS1 exerts a pivotal role in conducting signals from the insulin and insulin-like growth factor-1 (IGF-1) receptors to PI3K / Akt and Erk/ MAP kinase pathways. Insulin receptor substrate 2 (IRS-2) exerts a pivotal role in signal transduction mediated by the binding between insulin and its receptor. In pathological conditions, low expressions of IRS-1 and IRS-2 in fat cells predict IR. To evaluate IRS-1 and IRS-2 mRNA expressions in mature adipocytes upon tested conditions, Real-Time PCR was performed. IR induced a decrease in both receptor expressions (TNF-a and TNF-a+INS) compared to control conditions. On the other hand, as shown in Fig. 2D, E, CXCR1/2 antagonism increased IRS-1 and 2 expressions (TNF-a+LAD and TNF-a +LAD + INS) with respect to IR conditions.

**Fig. 3:**
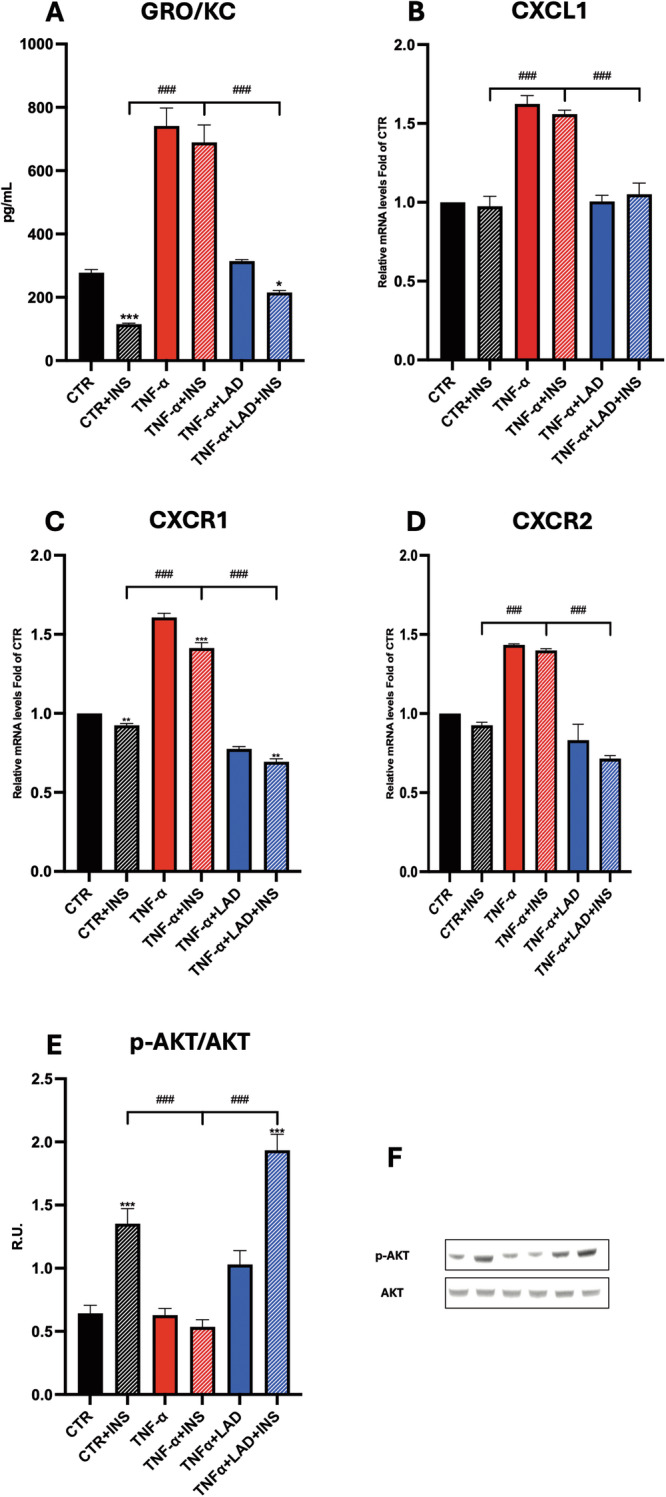
Analysis of IR underlying pathways in adipocytes. **A** ELISA assay for the quantification of IL-8, **B** IL-8, **C** CXCR1, **D** CXCR2 expressions by Real-Time PCR, **E** Western Blotting for p-AKT and AKT protein levels and relative densitometric analysis in adipocytes upon different conditions. A representative figure is shown. Data are mean ± SE of 3 different experiments; One way ANOVA. ^#^*p* *<* 0.05, ^##^*p* *<* 0.005, ^###^*p* *<* 0.0005 vs. the other conditions with the same INS stimulation (*N* *=* 3); *p *<* 0.05, **p *<* 0.005, ***p *<* 0.0005 vs. their respective condition without INS stimulation (*N* *=* 3).

CXCL1 secretion was significantly increased in IR conditions (TNF-a and TNF-a +INS, 741.1 ± 56.6 and 689.4 ± 55.1 pg/ml, respectively) compared to the respective control conditions (277.7 ± 9.8 and 115.3 ± 3.0 pg/ml, respectively). Interestingly, inhibition of CXCR1/2 decreased CXCL1 secretion (TNF-a +LAD and TNF-a +LAD + INS, 314.6 ± 4.4 and 215.3 ± 6.3 pg/ml, respectively) (Fig. 3A). These results were confirmed by gene expression analysis for CXCL1 (Fig. 3B). CXCR1 and CXCR2 mRNA expression levels were increased in IR conditions (TNF-a and TNF-a +INS) compared to the control conditions, while LAD decreased their expressions (TNF-a +LAD and TNF-a +LAD + INS) (Fig. 3C, D). These results suggest the activation of a negative feedback mechanism due to inhibition of CXCR1/2 receptors. The PI3K/Akt signaling pathway regulates numerous biological functions including cell proliferation, differentiation, metabolism, and cytoskeletal reorganization [26]. Due to its characteristics, this pathway is associated with several diseases including obesity and diabetes. When excessive energy intake occurs, the PI3K/Akt pathway is hindered. Therefore, activation of the PI3K/Akt pathway alleviates obesity and IR [27]. Interestingly, in our experimental conditions, we observed a decrease in Akt phosphorylation in IR model (Fig. 3E, F), which was reverted by CXCR1/2 inhibition.

### Metabolic profile of IR adipocytes

**Fig. 4:**
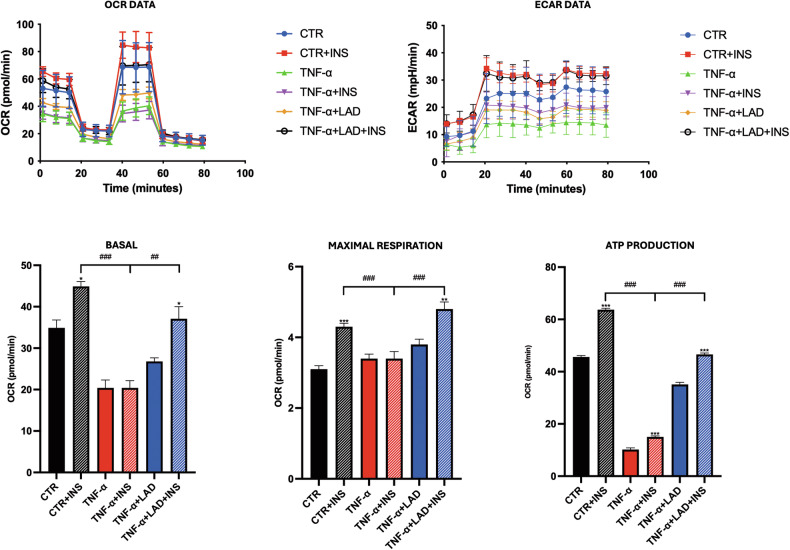
Metabolic profile of IR adipocytes. Seahorse Mitostress assay of IR adipocytes upon different conditions are shown. Data are mean ± SE of 3 different experiments; One way ANOVA. ^#^*p* < 0.05, ^##^*p* < 0.005^, ###^*p* < 0.0005 vs. the other conditions with the same INS stimulation (*N* = 3); *p < 0.05, **p < 0.005, ***p < 0.0005 vs. their respective condition without INS stimulation (*N* = 3).

We subsequently examined the biological consequences of IR in our model by analyzing the oxygen consumption rate (OCR) and glycolytic flux (assessed via extracellular acidification rate, ECAR) using a Seahorse Analyzer. As reported in Fig. 4, TNF-α challenge (both TNF-α and TNF-α + INS) resulted in a marked decrease in OCR, basal respiration, maximal respiration and ATP production in IR adipocytes compared to the control group (CTR). In contrast, the inhibition of CXCR1/2 receptors (LAD) significantly enhanced the bioenergetic profile. Additionally, ECAR measurements showed that cells treated with TNF-α exhibited a lower glycolytic rate compared to control cells. Importantly, the presence of LAD improved this parameter as well, indicating that receptor inhibition can positively impact the bioenergetic profile under IR conditions (Fig. 4).

### Hepatocytes: analysis of the pathways involved in IR

**Fig. 5:**
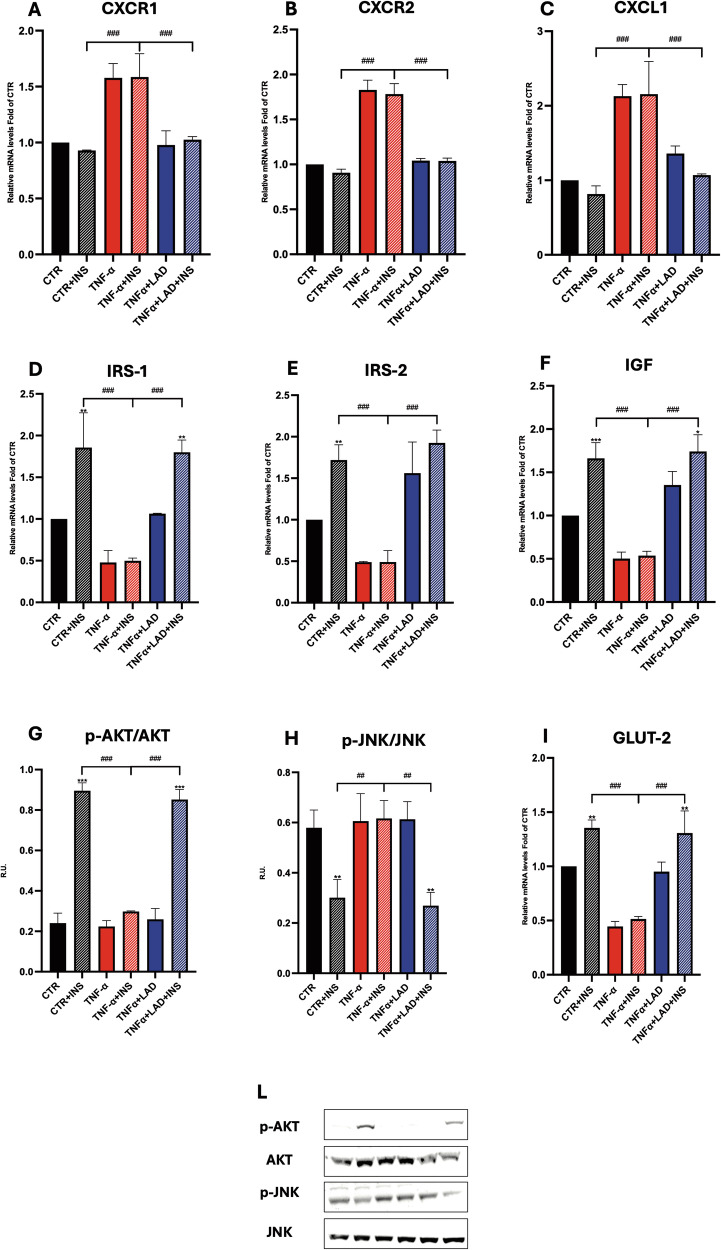
Analysis of the IR underlying pathways in hepatocytes. **A** CXCR1, **B** CXCR2 and **C** CXCL1 expressions by Real-Time PCR in hepatocytes upon different conditions. **D** IRS-1 and **E** IRS-2 expressions by Real-Time PCR in hepatocytes upon different conditions. Western Blotting for (**G**) p-AKT and AKT and (**H**) p-JNK/JNK protein levels and relative densitometric analysis in hepatocytes upon different conditions. A representative figure is shown. **F** IGF and **I** GLUT-2 expressions by Real-Time PCR in hepatocytes upon different conditions. **L** Representative WB figures. Data are mean ± SE of 3 different experiments; One way ANOVA. ^#^*p* < 0.05, ^##^*p* < 0.005, ^###^*p* < 0.0005 vs. the other conditions with the same INS stimulation (*N* = 3); *p < 0.05, **p < 0.005, ***p < 0.0005 vs. their respective condition without INS stimulation (*N* = 3).

We first focused on the expression of CXCR1 and CXCR2 in hepatocytes by Real-Time PCR. Results showed that CXCR1 expression is increased in IR conditions (TNF-a and TNF-a+INS) compared to control conditions (CTR and CTR + INS). Notably, the antagonist decreased CXCR1 expression (TNF-a+LAD and TNF-a+LAD + INS) (Fig. 5A). The same behavior was observed concerning CXCR2 expression (Fig. 5B).

The presence of inflammatory conditions was confirmed by the analysis of CXCL1 gene expression that resulted up-regulated upon IR conditions, while Ladarixin, the dual inhibition of CXCR1/2, was able to counteract this effect (Fig. 5C).

To evaluate the effect of CXCR1 and 2 inhibition on insulin signaling in murine hepatocytes, the expression of IRS1 and IRS2 receptors by Real-Time PCR was analyzed. Interestingly, IRS1 and 2 levels resulted significantly downregulated in the IR model, while ladarixin was able to counteract this effect, thus ameliorating the insulin resistance condition (Fig. 5). Due to the known involvement of the phosphorylated form of Akt and JNK in insulin resistance [28], these proteins were analyzed by western blotting analysis. Under insulin treatment (CTR + INS), p-Akt was significantly up-regulated, paralleled by a significant decrease of p-JNK. The insulin resistance model (TNF-a and TNF-a+INS) showed an opposite behavior, i.e., a significant decrease of p-Akt and a significant increase of p-JNK. Interestingly, in our experimental conditions, CXCR1/2 inhibition in the insulin resistance model (TNF-a+LAD + INS) showed a behavior close to control (CTR + INS), thus suggesting CXCR1/2-mediated ameliorations in the pathways related to glucose uptake and insulin resistance (Fig. 5G, H).

IGF expression and GLUT2 expression were then analyzed upon different conditions by Real-time PCR. A decreased IGF expression was observed in the IR model (TNF-a and TNF-a+INS) while ladarixin restored its expression to the control condition.An increase in IGF expression ws also observed upon INS stimulation, thus suggesting insulin sensitivity retrieval. Regarding GLUT2, the glucose transporter was not significantly modulated by INS stimulation (TNF-a+INS), while CXCR1/2 inhibition (TNF-a+LAD + INS) fully restores basal GLUT2 expression and sensitivity to insulin stimulations (Fig. 5F, I).

### Metabolic profile analysis in IR hepatocytes

**Fig. 6:**
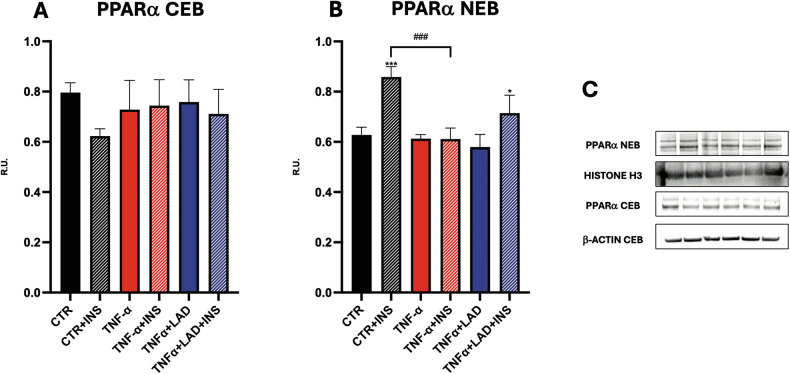
Metabolism in IR hepatocytes. **A**, **B** Western Blot analysis for nuclear and cytoplasmic PPARα in hepatocytes. **C** Representative WB figures are shown. Data are mean ± SE of 3 different experiments; One way ANOVA. ^#^*p* < 0.05, ^##^*p* < 0.005, ^###^*p* < 0.0005 vs. the other conditions with the same INS stimulation (*N* = 3); *p < 0.05, **p < 0.005, ***p < 0.0005 vs. their respective condition without INS stimulation (*N* = 3).

PPARα is involved in hepatic lipid and glucose metabolism and exhibits anti-steatotic effects but also anti-inflammatory activity at the hepatic level [29]. Accordingly, in our experimental conditions, a significant increase in nuclear PPARα was observed in response to insulin stimulation, while in IR conditions this insulin response was abrogated. Notably, ladarixin was able to stimulate the translocation of PPARα at the nuclear level, thus suggesting an improvement in insulin response (Fig. 6).

**Fig. 7:**
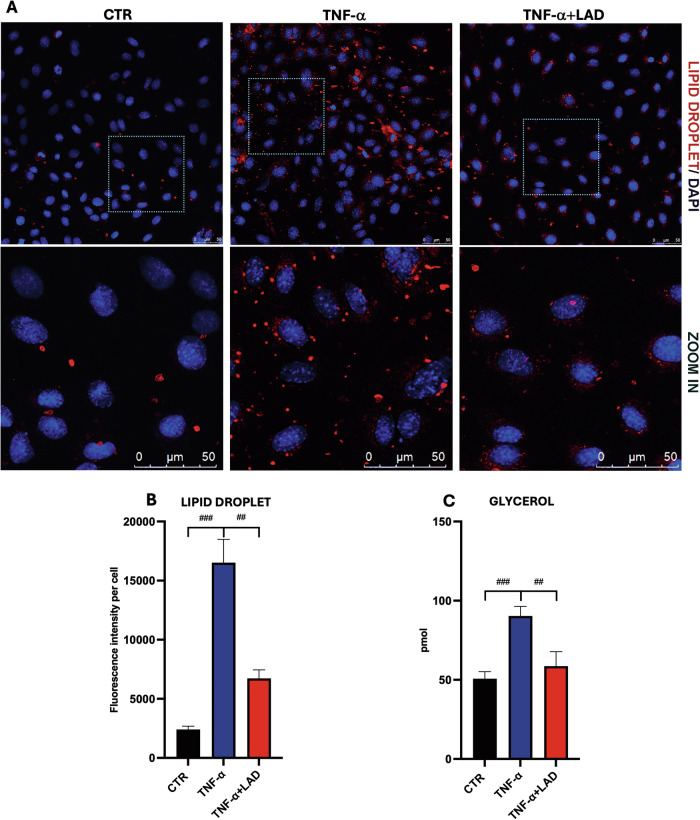
Lipid droplets and glycerol content in IR hepatocytes. **A** Nile Red staining marking LD in hepatocytes untreated (CTR), treated with TNF-a and TNF-a+LAD. Bar=50 µm, **B** LD intensity per cell count, **C** Lipolysis assay in IR-hepatocytes. Strippled blue square: Zoom in. Data are mean ± SE of 3 different experiments; One way ANOVA. ^#^*p* < 0.05, ^##^*p* < 0.005, ^###^*p* < 0.0005 vs. the other conditions with the same INS stimulation (*N* = 3); *p < 0.05, **p < 0.005, ***p < 0.0005 vs. their respective condition without INS stimulation (*N* = 3).

Hepatocytes are parenchymal cells of the liver responsible for mobilizing lipids for energy and storing excess lipids in the form of lipid droplets (LDs), therefore compelling the liver the primary organ accountable for lipid homeostasis. Hepatocellular accumulation of excess LDs is called hepatic steatosis [30]. Thus, in the light of a PPARα activation observed upon ladarixin treatment in IR conditions, we analyzed the LD formation in hepatic cells marking them with Nile Red staining in CTR, TNF-a, and TNF-a +LAD by fluorescence microscopy. TNF induced intracellular LD formation and accumulation while the copresence of LAD was able to counteract this effect (Fig. 7A). These results were supported by a high-sensitivity lipolysis assay. In our model an increase in lipolysis upon TNF-a was observed, thus reflecting the development of IR, while upon LAD this effect was strongly counteracted (Fig. 7B). The conditions with insulin stimulation were omitted due to the short time of pulse that it is not able to allow lipid mobilization. These results were also confirmed by LD count, in which the inhibition of CXCR1/2 significantly reduced LDs formation (Fig. 7C).

**Fig. 8:**
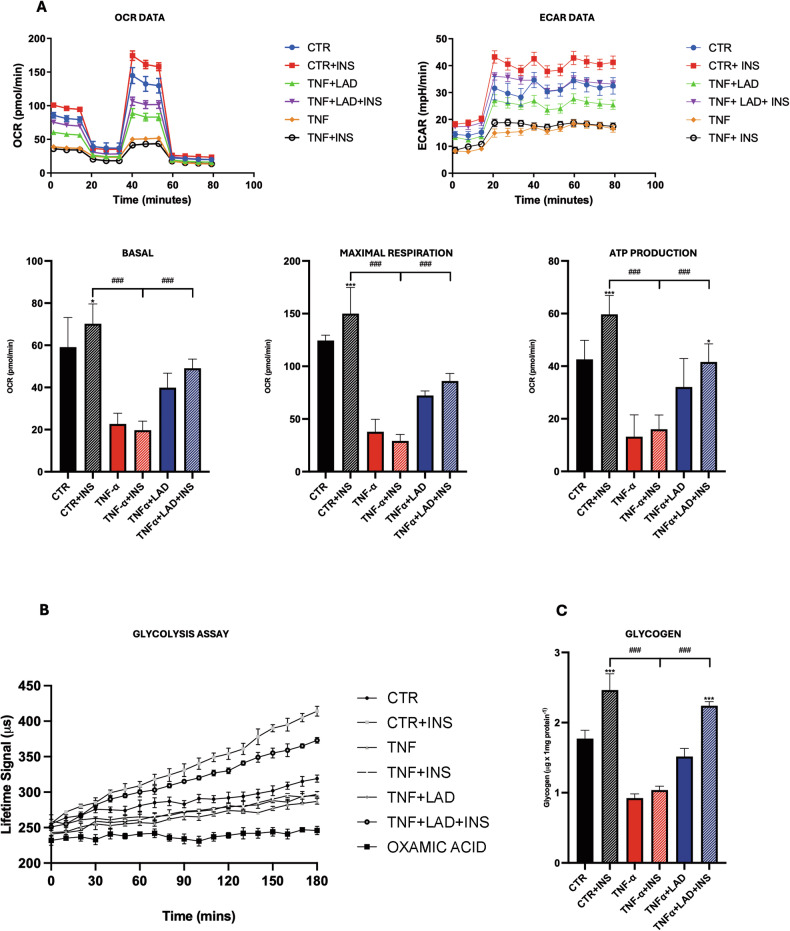
Bioenergetic profile of IR hepatocytes. **A** Seahorse Mitostress assay and **B** Glycolysis assay in hepatocytes upon different conditions, data were expressed as Lifetime Signal (µs) (Significativity in a Suppl table), **C** Glycogen concentration in hepatocytes upon different conditions. Dataare mean ± SE of 3 different experiments; One way ANOVA. ^#^*p* < 0.05, ^##^*p* < 0.005, ^###^*p* < 0.0005 vs. the other conditions with the same INS stimulation (*N* = 3); *p < 0.05, **p < 0.005, ***p < 0.0005 vs. their respective condition without INS stimulation (*N* = 3).

We then focused on the biological implications of IR in our model studying the oxygen consumption rate (OCR) and the substrate level phosphorylation via glycolysis (i.e., via extracellular acidification rate (ECAR) using Seahorse Analyzer. As shown in the Fig. 8, TNF-a (TNF-a and TNF-a+INS) lead to a significant reduction of OCR, basal and maximal respiration, and ATP production in IR hepatocyte cells when compared to the control, while the inhibition of CXCR1/2 was able to improve the bioenergetic profile. The ECAR in cells exposed to TNF-a was lower compared to control cells and also in this case the presence of LAD improved the situation, thus suggesting that the receptor inhibition can ameliorate the bioenergetic profile occurring upon IR (Fig. 8A).

Seahorse bioenergetic results were supported by a glycolysis assay. Specifically, the typical lifetime profile of Glycolysis Assay for FL83B upon different treatments is reported. Oxamic acid was used as negative control (as it is known to decrease ECAR). CTR hepatocyte cells showed a profile higher than oxamic acid. As expected, insulin stimulates glycolysis while in the TNF-a condition, we did not observe any variation. On the other hand, LAD was able to counteract this effect; indeed, it increased the extracellular acidification rate indicating a restoration of insulin sensitivity (Fig. 8B).

Glycogen deposition in peripheral tissues and the liver is a physiological response in mammals to increased glycemia that occurs after a meal (Ferrer et al., 2003). Insulin brings a series of signal transduction pathways and dephosphorylates glycogen synthase (GS), which activates this enzyme thus promoting glycogen synthesis and reducing the blood glucose concentration under normal conditions (Saltiel & Kahn, 2001). Indeed, in our experimental conditions, in mouse liver FL83B cells, exposure to insulin significantly increased glycogen content in the control group, while in IR hepatocytes (TNF-a) the presence of insulin did not lead to any changes (TNF-a+INS). Notably, the inhibition of CXCR1/2 increased glycogen content in liver cells, upon insulin the enzyme activity was 2.2 ± 0.13 μg/mg protein, reflecting increases of about 50% and 120% as compared with the TNF-a group (Fig. 8C).

## Discussion

Recent research has expanded our understanding of chemokine signaling to encompass various non-inflammatory tissues. One emerging area of interest lies in the role of chemokines in the regulation of skeletal muscle function and repair [31]. Moreover, chemokine signaling has been implicated in regulating bone metabolism and homeostasis. Chemokines and their receptors are expressed in osteoblasts, osteoclasts, and bone marrow stromal cells, influencing bone remodeling processes [32]. Furthermore, emerging evidence suggests a role for chemokines in modulating adipose tissue biology and metabolism. Chemokine receptors are expressed on adipocytes and stromal cells within adipose tissue, implicating them in adipogenesis, inflammation, and insulin resistance associated with obesity [33]. Emerging studies have shed light on the intricate interplay of chemokines in non-inflammatory tissues, particularly within the central nervous system (CNS) and peripheral nerves. Beyond the CNS, emerging research has also implicated chemokines in regulating peripheral nerve function and repair processes [34]. Overall, these recent findings underscore the multifaceted roles of chemokine signaling in non-inflammatory tissues and highlight exciting avenues for further exploration in understanding their contributions to normal physiology and disease pathology. Chemokine signaling also profoundly influences the onset and development of metabolic diseases. Chemokines are now recognized as crucial regulators of metabolic processes in various tissues, including adipose tissue, liver, muscle, and pancreas [35]. Dysregulation of chemokine signaling has been implicated in the pathogenesis of metabolic diseases such as obesity, insulin resistance, T2D, and non-alcoholic fatty liver disease (NAFLD) [15]. Chemokines and their receptors are expressed in adipocytes, hepatocytes, and pancreatic β-cells, where they modulate lipid metabolism, insulin sensitivity, and glucose homeostasis [36].

Inflammatory chemokines, such CXCL8 (chemokine ligand 8), are elevated in obesity and insulin resistance and contribute to the recruitment of immune cells into metabolic tissues, fostering chronic low-grade inflammation [37]. This inflammatory milieu disrupts insulin signaling pathways, promoting insulin resistance and impairing glucose uptake in peripheral tissues [38]. Moreover, chemokines can directly influence adipocyte function, altering adipokine secretion and adipose tissue remodeling, further exacerbating metabolic dysfunction [39]. In addition to their inflammatory actions, chemokines also play pivotal roles in non-inflammatory cells involved in metabolic regulation. Furthermore, chemokine signaling within pancreatic islets influences β-cell survival, proliferation, and insulin secretion, impacting glucose metabolism and T2D development [40].

Understanding the intricate interplay of chemokine signaling in both inflammatory and non-inflammatory cells is essential for unraveling the complex pathophysiology of metabolic diseases. Targeting chemokine pathways may offer promising therapeutic strategies for mitigating metabolic dysfunction and preventing the progression of metabolic diseases.

In this context, we utilized a TNF-α-based cellular model to induce insulin resistance (IR) due to its established role as a pro-inflammatory cytokine that disrupts insulin signaling. This model, grounded in our previous studies [18], aims to replicate the inflammatory environment seen in metabolic disorders. However, we acknowledge that this model, while effective for studying acute inflammatory responses, presents certain limitations. Specifically, it does not fully capture the complex interaction of metabolic signals, such as lipids and glucose, that naturally lead to IR. Moreover, the focus on TNF-α may overlook the chronic nature of insulin resistance observed in vivo. Despite these constraints, this approach provides valuable insights into the inflammatory mechanisms underlying IR in adipocytes and hepatocytes.

Based on these findings, in our study, we examined the significance of chemokine signaling, particularly focusing on the CXCR1/2 pathway, in T2D adipose tissue and liver concerning TNF-a induced IR.

Mitochondrial dysfunction plays a crucial role in IR, as mitochondria are central to cellular energy homeostasis. In IR conditions, excessive lipid accumulation and chronic inflammation, particularly mediated by TNF-α, lead to oxidative stress and impaired mitochondrial function [41]. This includes a reduction in mitochondrial oxidative capacity, altered mitochondrial dynamics, and increased production of reactive oxygen species (ROS). These processes exacerbate insulin resistance by further impairing glucose metabolism in both adipocytes and hepatocytes. Importantly, targeting inflammatory pathways, such as TNF-α and CXCR1/2, may help restore mitochondrial function by reducing ROS production and improving insulin sensitivity [41]. Different studies have shown that improving mitochondrial health can directly alleviate insulin resistance, highlighting the importance of addressing both inflammation and mitochondrial dysfunction in therapeutic strategies for type 2 diabetes [42].

Our findings underscored notable alterations within insulin signaling pathways in adipocytes and hepatocytes, hinting at the potential of CXCR1/2 in modulating insulin sensitivity and resistance [43]. Our findings support the hypothesis that chronic inhibition of CXCR2 signals may delay the onset and prevent the development of TNF-a induced insulin resistance and related complications [43].

The effects of CXCR1/2 inhibition demonstrated by siRNA, pharmacological inhibition, or neutralization on insulin signaling both in adipocytes and hepatocytes were analyzed. The silencing or inhibition of the chemokine receptors affected IR parameters such as glucose uptake and IRS expression. IR reduced insulin-stimulated Akt phosphorylation, while the pharmacological inhibition of CXCR1/2 abolished this TNF-α induced effect. In the context of glycogen, insulin plays a crucial role in promoting glycogen synthesis. Glycogen is a form of glucose storage in the body, primarily in the liver and muscle tissues. When insulin resistance occurs, this process can be impaired. Glycogen synthesis is a downstream event in the liver dependent on insulin and Akt. This event is confirmed in our experimental conditions, where glycogen content is significantly decreased and restored under CXCR1/2 inhibition in IR conditions. In summary, IR can disrupt the normal processes of glycogen synthesis and glucose regulation in the body in dependence on CXCR1/2 activation. These results may indicate that interfering with CXCR1/2 signaling could diminish the insulin-resistant state induced by TNF-α.

The link between PPARα and CXCR1/CXCR2 in the liver and adipose tissue has been investigated in several studies, shedding light on their roles in metabolic regulation, inflammation, and lipid metabolism. PPARα is a key regulator of lipid metabolism and inflammation in the liver. CXCR1 and CXCR2, on the other hand, are involved in the regulation of inflammatory responses. A previous study explored the role of PPARα in liver inflammation and fibrosis. The study demonstrated that PPARα activation attenuated liver fibrosis by reducing the expression of pro-inflammatory cytokines and chemokines, including CXCR1 and CXCR2, thus highlighting the potential link between PPARα and CXCR1/2 in liver inflammation and fibrosis [44]. Another study investigated the role of PPARα in adipose tissue inflammation. The study revealed that PPARα activation suppressed adipose tissue inflammation by reducing the expression of CXCR1 and CXCR2, leading to improved insulin sensitivity and reduced adipose tissue inflammation [45]. Moreover, it has been demonstrated that PPARα activation leads to the upregulation of genes involved in fatty acid oxidation, such as CPT1A (carnitine palmitoyltransferase 1A), ACOX1 (acyl-CoA oxidase 1), and MCAD (medium-chain acyl-CoA dehydrogenase). These genes are crucial for the breakdown of fatty acids in the mitochondria and peroxisomes, promoting energy production and reducing lipid accumulation [46]. These studies provide evidence of the link between PPARα and CXCR1/2 in the regulation of inflammation, lipid metabolism, and metabolic disorders in both the liver and adipose tissue, thus supporting our findings on the role of CXCR1/2 in metabolic alteration and inflammation observed in our experimental conditions.

In summary, the interplay between CXCR1/2 and TNF-α signaling pathways is complex and multifaceted, involving modulation of inflammatory responses and insulin sensitivity. By investigating the impact of CXCR1/2 inhibition on TNF-α induced insulin resistance, this study seeks to provide insights into the potential therapeutic targeting of chemokine receptors to improve insulin sensitivity. However, it is crucial to acknowledge potential limitations associated with long-term CXCR1/2 inhibition, particularly regarding susceptibility to infections. While our study provides valuable insights into the role of CXCR1/2 signaling in metabolic diseases, ongoing research is essential to address remaining questions and potential safety concerns associated with therapeutic interventions targeting these receptors. Future studies will also be devoted to dissecting the interaction between IGF and GLUT2 in these models, which are significant characters in regulating glucose metabolism and homeostasis. Even if CXCR1/2 inhibition therapies are really promising for treating various disorders, it would be crucial for future clinical applications to evaluate the possible unwanted side effects, such as neutropenia, increased risk infections and gastrointestinal problems.

## Material and methods

### Insulin-resistant adipocytes

3T3-L1 murine preadipocytes cell line was bought from ATCC (American Type Culture Collection, Manassas, VA, USA), and cultured in DMEM with 10% newborn calf serum (NCS) at 37 °C in a 5% CO_2_ atmosphere until confluence. At 2 days after full confluence, cells were differentiated using DMEM (10% FBS) supplemented with 0.5 mM isobutylmethylxanthine (IBMX), 1 μM dexamethasone, 1 μg/ml insulin for 48 h, and then for 2 days in DMEM (10% FBS) containing 1 μg/ml insulin alone following manufacturer’s protocols (Sigma-Aldrich, St. Louis, MO, USA) [18]. Cells were maintained and refed every 2 days with DMEM containing 10% FBS. After 22 days, more than 80% of the cells exhibited the adipocyte phenotype with large lipid droplets in the cytoplasm.

The IR condition was obtained by incubating the differentiated cells with TNF-a 50 ng/mL for 24 h. Induction of IR was proven by the ability of insulin to stimulate glucose uptake. Once the model was established, adipocytes were treated for 24 h with Ladarixin at 10 μM, a concentration that has already shown to be safe and effective in previous in vitro study [18], then cellular biochemical parameters were analyzed in the presence or absence of stimulation with insulin (1 μM) for 30 min (short-term insulin). To activate the insulin pathway, insulin was added at the end of 24 h at 1 µM concentration for 20 min. Different treatments were used to study the effects on TNF-induced IR adipocytes.Adipocytes cells were divided into 6 groups:

1. Control cells (CTR)
2. Control cells challenged with a short-term exposure to insulin (CTR + INS)
3. TNF-a treated cells (TNF)
4. TNF-a treated cells subsequently challenged with a short-term exposure to insulin (TNF-a+INS)
5. TNF-a treated cells upon Ladarixin treatment (TNF-a+LAD)
6. TNF-a treated cells upon Ladarixin treatment subsequently challenged with a short-term exposure to insulin (TNF-a+LAD + INS)

### Insulin-resistant hepatocytes

Mouse liver cell line FL83B CRL-2390™ was purchased from ATCC and cultured following the manufacturer’s protocols. Specifically, cells were incubated in F12-K Medium containing 10% FBS (all products from ATCC) in Petri dishes at 37 °C and 5% CO_2_. After 2 days from seeding, the IR condition was obtained by incubating the cells with TNF-α 20 ng/mL for 24 h with and without 10 μM of Ladarixin. To activate the insulin pathway, insulin was added at the end of 24 h at 1 µM concentration for 30 min. Different treatments were used to study the effects on TNF-induced IR hepatocytes.

Hepatocytes cells were divided into 6 groups:

1. Control cells (CTR)
2. Control cells challenged with a short-term exposure to insulin (CTR + INS)
3. TNF-a treated cells (TNF)
4. TNF-a treated cells subsequently challenged with a short-term exposure to insulin (TNF-a+INS)
5. TNF-a treated cells upon Ladarixin treatment (TNF-a+LAD)
6. TNF-a treated cells upon Ladarixin treatment subsequently challenged with a short-term exposure to insulin (TNF-a+LAD + INS)

### CXCR1/2 silencing

siRNA knockdown was performed using Lipofectamine™ RNAiMAX Transfection Reagent (ThermoFisher Scientific, Waltham, MA, USA) and CXCR1 (ID s105602) and/or CXCR2 (ID s64085) specific siRNA duplexes (ThermoFisher Scientific) according to the manufacturer’s instructions. FL38B hepatocyte and differentiated 3T3-L1-adipocytes were seeded to 1 × 10^4^ cells/cm^2^ in a 96 well plate overnight and trasfected with 1 pmol of CXCR1 and/or CXCR2 siRNA duplexes, or 1 pmol of negative control siRNA duplexes. Knockdown of CXCR1/CXCR2 expression was confirmed after 72 h by evaluating CXCR1 and CXCR2 gene expression via qRT-PCR.

### Neutralization with antibodies

Mature adipocytes and hepatocytes were treated with neutralizing antibodies against CXCR1 and CXCR2. Cells were incubated with anti-CXCR1 (CD Creative Diagnostics, Shirley, NY, USA) and anti-CXCR2 (GeneTex, Irvine, CA, USA) antibodies (according to the manufacturer’s protocols) for 24 h at 37 °C in a humidified atmosphere containing 5% CO_2_. Control cells were treated with an equal concentration of isotype-matched control antibodies.

### Glucose uptake

To monitor the uptake of glucose, mature adipocytes and hepatocytes upon different treatments were incubated with the fluorescent tracer 2-NBDG (2-Deoxy-2-[(7-nitro-2,1,3-benzoxadiazol-4-yl)amino]-D-glucose; Sigma-Aldrich) as previously described [18]. Data are reported as 2-NBDG fluorescence intensity.

### Nile Red Staining

Nile Red is a commonly used neutral lipid stain to detect the adipogenesis of different types of cultured cells. Briefly, after culturing and treating as previously described, cells were washed with PBS and incubated with Nile Red (final concentration 50 ng/mL) for 30 min, then washed and observed at fluorescence microscopy (Olympus, Leica, Germany).

### Western blotting

Control and treated hepatocytes and adipocytes were collected and lysed in ice-cold RIPA buffer with freshly added protease and phosphatase inhibitor cocktails (Thermo Fisher Scientific). 30 μg of proteins were loaded on Bolt Bis-Tris Glycine gradient precast gel (Invitrogen, Carlsbad, CA, USA) and transferred onto a PVDF membrane using a power blotter-semi-dry transfer system (Thermo Fisher Scientific). Non-specific binding sites were blocked by Blocking Buffer (Invitrogen) for 10 min at RT. Membranes were then incubated overnight at 4 °C with the following primary antibodies, diluted in blocking buffer: anti-Akt 1:1000 (4691 s Cell Signaling Technology, Danvers, MA, USA), anti-pAkt 1:1000 (4060 s Cell Signaling Technology, Danvers, MA, USA), anti-GLUT4 1:1000 (2213 s Cell Signaling Technology, Danvers, MA, USA), anti-JNK 1:200 (9252 s Cell Signaling Technology, Danvers, MA, USA), anti-PPARα 1:250, anti-pJNK 1:200 (9255 s Cell Signaling Technology, Danvers, MA, USA), and conjugated Actin 1:20000 (5125S, Cell Signaling Technology, Danvers, MA, USA). As secondary antibodies, 1:20,000 peroxidase-conjugated anti-rabbit or anti-mouse IgG were used. Immunoreactive bands were visualized by luminol (Thermo Fisher Scientific), according to the manufacturer’s protocols, and visualized at iBright digital system (Thermo Fisher Scientific). The relative densities of the immunoreactive bands were determined and normalized to tubulin or actin or histone H3 or their respective total forms using Fiji software. Values were reported as relative units.

### Subcellular protein fractionation assay

For the subcellular protein fractionation, cells were cultured as described above, collected and incubated with provided buffer to extract the cytoplasmatic, membrane nuclear components as previously published [18] and then performed the Western blotting analyses as described above.

### Real-Time PCR

Total RNA was extracted by Miniprep column Qiagen according to the manufacturer’s instructions. To improve the extraction procedure of adipocytes, detergents (Deoxycholate-Nonidet P40) were added. The total RNA concentration was determined in RNAse-free water using Nanodrop, while the concentration was determined using Qubit Fluorometer 3.0 (Thermo Fisher Scientific). 1 μg of total RNA was reverse transcribed using a 5X All-In-One RT MasterMix (Applied Biological Materials, Richmond, BC, Canada) into cDNA using Thermo-block (Eppendorf AG, Hamburg, Germany). Finally, the real-time PCR was carried out on ABI 7300HT sequence detection system (Applied Biosystems, Waltham, MA, USA), containing 2X TaqMan Gene Expression Master Mix (Invitrogen), DEPC water and 5 μL of cDNA and 1 μL of the following primers. Prime Time qPCR Assays: mouse CXCL1, CXCR1, CXCR2, IRS1, IRS2, GLUT2, IGF, GLUT4, Adiponectin were purchased from ThermoScientific. Triplicate samples were run for each gene. The reference gene GAPDH was used as an internal control to normalize the expression of target genes. Relative expression levels were analyzed for each sample after normalization against the reference gene, using the ΔΔCt method for comparing relative fold expression differences [47, 48].

### SeaHorse analysis

Seahorse XF96e Extracellular Flux Analyzer (Agilent Technologies, CA, USA) to investigate the bioenergetic profiles of our IR in vitro models upon different conditions was used. Live-cell evaluations of oxygen consumption rate (OCR) and extracellular acidification rate (ECAR) were detected by the Mito Stress assay (Agilent). Cells were seeded at a density of 5 × 10^4^ cells/well (optimized to guarantee a proportional response of FCCP) on a Seahorse XF96 plates then treated as described above. Mito Stress assay was performed following manufacturer’s protocols, and the concentrations of the drugs were: 1 µM oligomycin, 1 µM FCCP, 0.5 µM rotenone/antimycin. For the normalization in port D, Hoechst 33,342 solution was added. Data were obtained using Wave software.

### Adiponectin mouse ELISA assay

To assess the released adiponectin in cell culture media from mature 3T3-L1 at the different conditions tested, an adiponectin mouse ELISA assay was used as previously described [18]. The results were read immediately at 450 nm in a plate reader. Data were expressed as ng/ml.

### GLUT4 mouse ELISA assay

The amount of glucose transporter type 4 (GLUT4) in adipocyte plasma membranes was evaluated using the ELISA KIT by MyBioSource, Inc (San Diego, CA, USA) (#MBS2022770) following the manufacturer’s instructions and as previously published [18]. Briefly, the cells were counted and lysed as suggested, then the Standard curve as well as the reagents were prepared and used following the assay procedure. The microplate provided in this kit has been pre-coated with an antibody specific to GLUT4. Standards or samples are then added to the appropriate microplate wells with a biotin-conjugated antibody specific to GLUT4. Next, Avidin conjugated to Horseradish Peroxidase (HRP) is added to each microplate well and incubated. After the TMB substrate solution is added, only those wells that contain GLUT4, biotin-conjugated antibody, and enzyme-conjugated Avidin will exhibit a color change. The enzyme-substrate reaction is terminated by the addition of sulphuric acid solution and the color change is measured spectrophotometrically at a wavelength of 450 nm ± 10 nm. The concentration of GLUT4 in the samples is then determined by comparing the O.D. of the samples to the standard curve. Data were presented as ng/ml.

### CXCL1/KC (mouse IL-8) ELISA assay

To evaluate the CXCL1/KC released from mature adipocytes in different conditions CXCL1/KC ELISA assay was performed following the manufacturer’s protocol (R&D System, Minneapolis, USA). Briefly, the plate was prepared the day before using Capture Antibody. Then, we followed the assay procedure preparing the standard curve and the samples, then the substrate solution was incubated for 20 min at room temperature, followed by the addition of stop solution. The optical density of each well was quickly determined using a microplate reader set to 450 nm. Data were expressed as pg/ml.

### Adipocyte lipolysis colorimetric assay

To evaluate the lipolysis, cells were grown and differentiated as previously described in a 48 wells plate. After 22 days, cells were washed two times with wash buffer and the wash buffer was replaced with lipolysis assay buffer. Then, isoproterenol (100 nM) was added to stimulate lipolysis 2 h and the medium was collected. 25 µl of media were added into 96well plate and the volume was adjusted with lipolysis Assay buffer to 50 µl. Standard curve was prepared following manufacturer’s protocol as well as reaction mix. The plate was incubated at room temperature for 30 min protected from light. The results were read at OD 570 nm in a plate reader. Data were expressed as glycerol content (nmol/well).

### High Sensitivity Lipolysis Assay for hepatocytes

To evaluate the lipolysis in hepatocytes, High Sensitivity Lipolysis assay was performed following manufacturer’s instruction (Sigma-Aldrich, #MAK215). Cells were grown in a black 96-well plate and treated as described above. Cells were washed two times with wash buffer, and then with lipolysis assay buffer. To stimulate lipolysis, isoproterenol (100 nM) was added and incubated for 8 h, and the medium was collected. 25 µL of media were added into 96 well-plates, and the volume was adjusted with lipolysis Assay buffer to 50 µL. The standard curve of glycerol and reaction mix were prepared. The plate was incubated at room temperature for 1 h protected from light. Lipolysis was determined by measuring a fluorescent product (λex = 535/ λem = 587 nm) proportional to the amount of glycerol present using the microplate reader Spark, Tecan and analyzed following instruction provided.

### Glycolysis assay

To evaluate the glycolysis, hepatocytes were grown in a black 96-well plate and treated as described above and glycolysis assay was performed following manufacturer’s instruction (Abcam, Waltham, USA #ab197244). While preparing all the required reagents, tested compound and Oxamic acid (negative control, decreases ECA) cells, a CO_2_ purge by incubating cells was performed in a CO_2_-free incubator at 37 °C with 95% humidity, approx. 3 h prior to performing the Glycolysis assay measurement.

We then washed cells with Respiration Buffer twice. Add 150 µL of Respiration Buffer to all wells containing cells. To the negative control 10 µL of oxamic acid solution (750 mM in ddH_2_O) to wells containing cells were added. 10 µL reconstituted Glycolysis Assay Reagent to each sample well were added and 10 µL of test compound (vehicle control and/or stock) were added to the wells. Plate was read at λex = 380/ λem = 615 nm using the microplate reader Spark, Tecan and analyzed following instruction provided.

### Glycogen assay

To evaluate the glycogen, hepatocytes were grown and treated as described above and glycogen assay was performed following manufacturer’s instruction (Abcam, #ab169558). For the sample preparation cells were rapidly homogenized on ice and then boiled for 10 mins to inactivate enzymes and then centrifuged. Supernatants were collected to perform the assay using 50 µg of proteins each well. In parallel, standards were prepared as indicated and both samples and standards were added to each well at the final volume of 50 µl. Hydrolysis enzyme was then added and incubated for 30 min at room temperature. Finally, reaction mix was added and incubated at room temperature for 30 mins. Plates were read at OD450 nm using a microplate reader Spark, Tecan and analyzed following instruction provided.

### Statistical analyses

All data were presented as mean ± SE. Data analyses were performed using GraphPad Prism 8 (GraphPad Software Inc., San Diego, CA, USA). For multiple comparisons one-way analysis of variance (ANOVA) followed by Tukey post-hoc tests was used. The level of significance was set at *p* < 0.05.

## Supplementary information

Supplementary Figure 1

supplementary FIG1 legends

Uncropped Western Blot

## Data Availability

Corresponding authors will be responsive to reasonable inquiries.
