# Supplementary figures and images for: TNFα-CXCR1/2 partners in crime in insulin resistance conditions
Source: Cell Death Discov. 2024 Dec 3;10:486. doi: 10.1038/s41420-024-02227-5 (PMC11615304; doi:10.1038/s41420-024-02227-5)

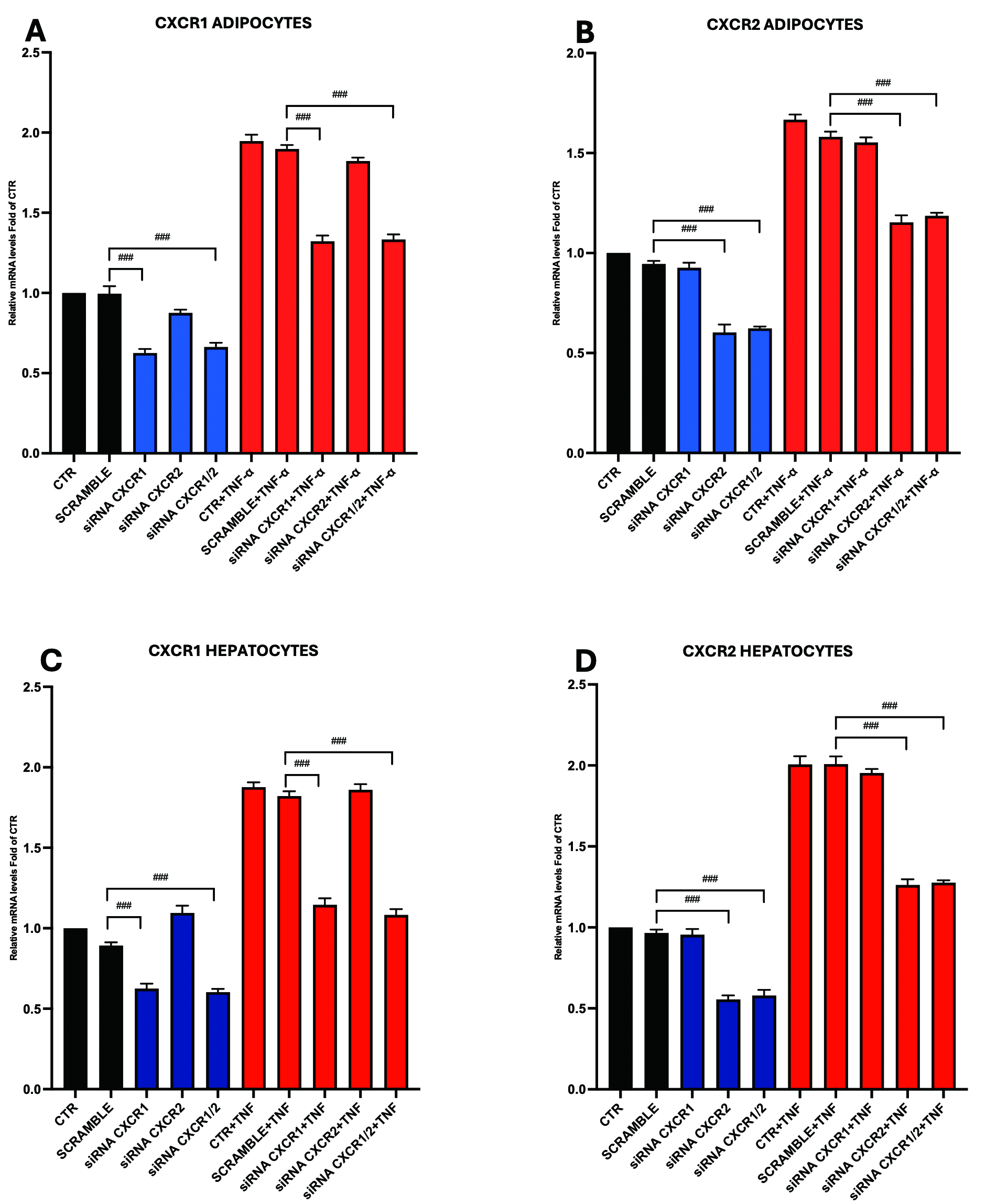

Supplement: Supplementary file 1 — Supplementary Figure 1 [file 41420_2024_2227_MOESM1_ESM.tif]
