# Supplementary material for: TNFα-CXCR1/2 partners in crime in insulin resistance conditions
Source: Cell Death Discov. 2024 Dec 3;10:486. doi: 10.1038/s41420-024-02227-5 (PMC11615304; doi:10.1038/s41420-024-02227-5)
Supplement: Supplementary file 2 — supplementary FIG1 legends [file 41420_2024_2227_MOESM2_ESM.docx]

**Suppl FIG1.** Real-Time PCR for (A) CXCR1and (B) CXCR2 in adipocytes and (C) CXCR1 and (D) CXCR2 in hepatocytes at different conditions. Data are mean ± SE of 3 different experiments; One way ANOVA. #p < 0.05, ##p < 0.005, ###p < 0.0005 vs their respective condition with SCRAMBLE (N = 3).
