# Supplementary material for: TNFα-CXCR1/2 partners in crime in insulin resistance conditions
Source: Cell Death Discov. 2024 Dec 3;10:486. doi: 10.1038/s41420-024-02227-5 (PMC11615304; doi:10.1038/s41420-024-02227-5)
Supplement: Supplementary file 3 — Uncropped Western Blot [file 41420_2024_2227_MOESM3_ESM.pdf]

FIGURE 3

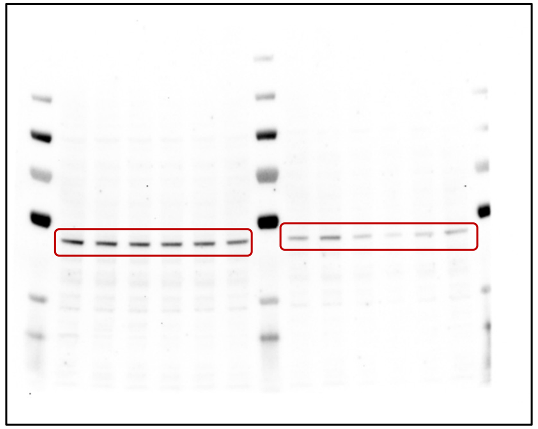

Legend:

1-CTR;
2-CTR + INS;
3- TNF-α;
4- TNF-α + INS;
5- TNF-α + LAD;
6- TNF-α + LAD + INS.

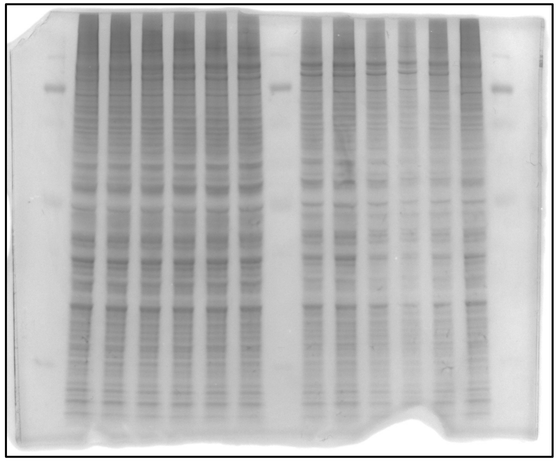

Legend:

1-CTR;
2-CTR + INS;
3- TNF-α;
4- TNF-α + INS;
5- TNF-α + LAD;
6- TNF-α + LAD + INS.

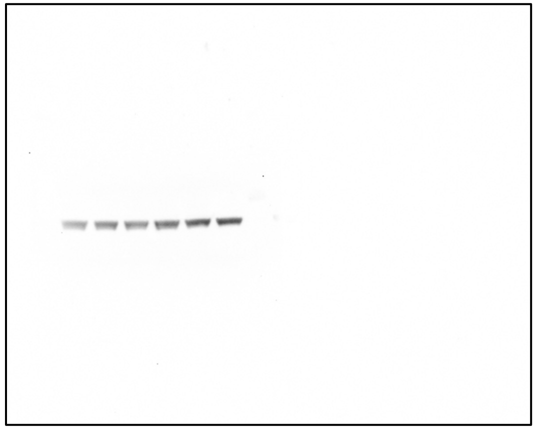

Legend:

1-CTR;
2-CTR + INS;
3- TNF-α;
4- TNF-α + INS;
5- TNF-α + LAD;
6- TNF-α + LAD + INS.

FIGURE 4

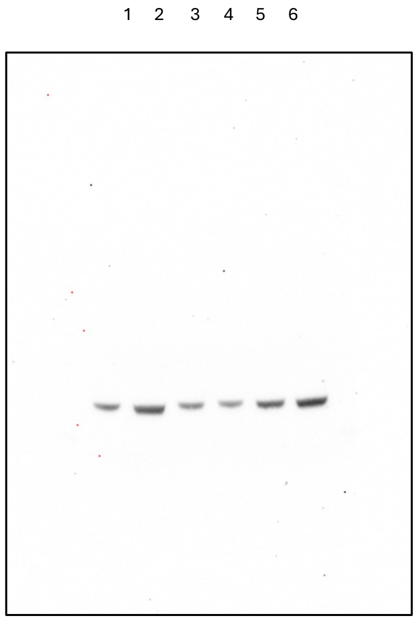

p-AKT

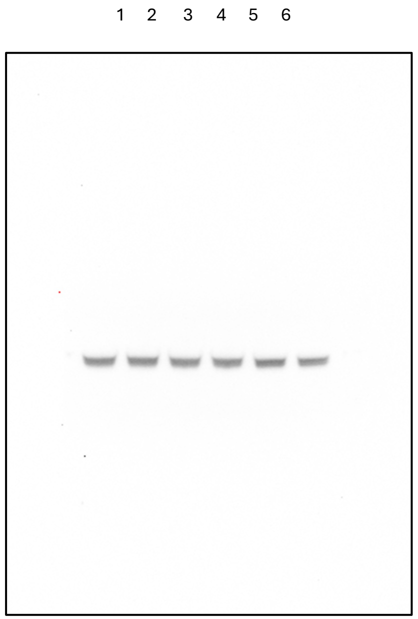

AKT

Legend:

1-CTR;
2-CTR + INS;
3- TNF-α;
4- TNF-α + INS;
5- TNF-α + LAD;
6- TNF-α + LAD + INS.

FIGURE 7

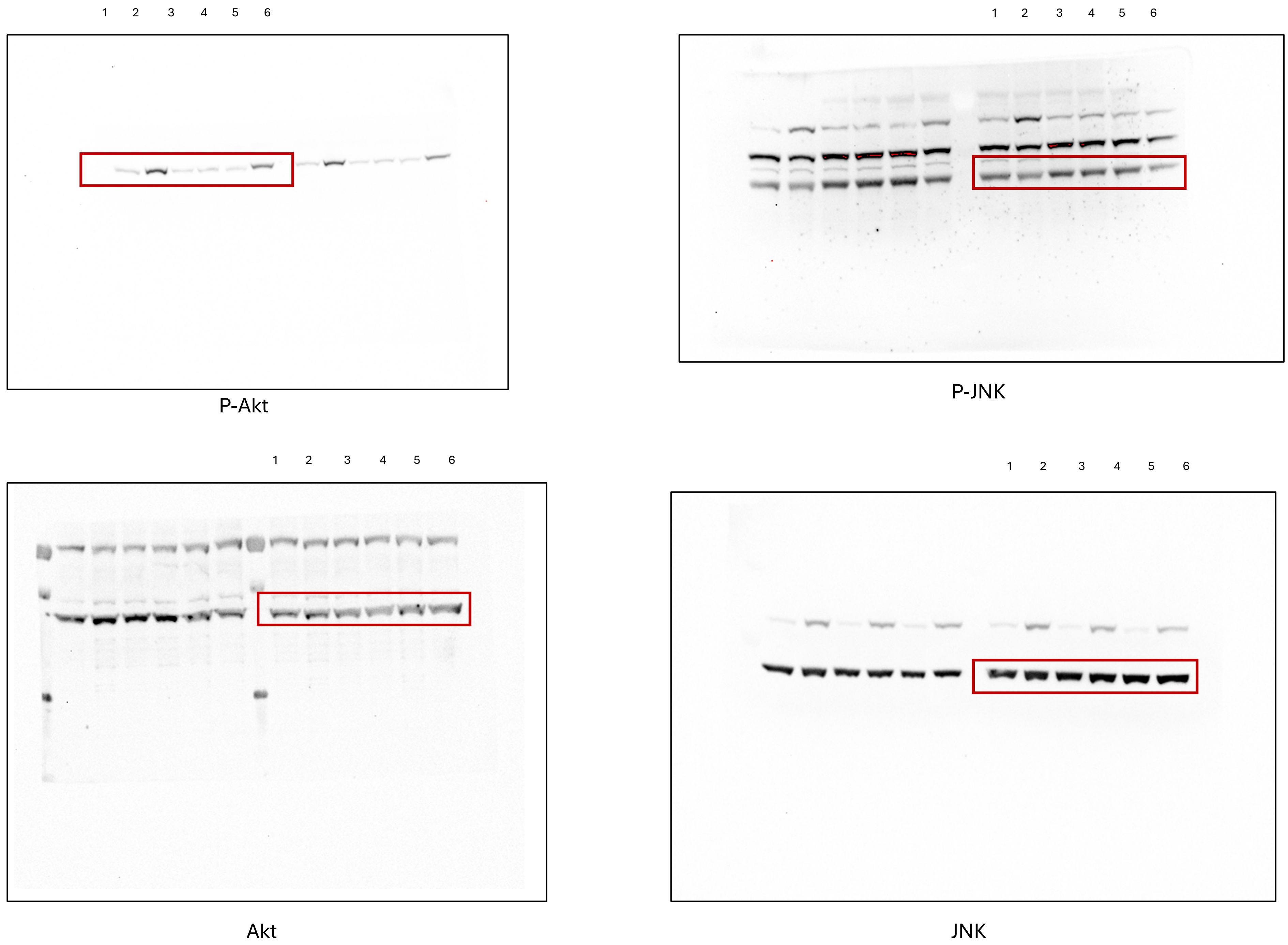

Legend:

1-CTR;
2-CTR + INS;
3- TNF-α;
4- TNF-α + INS;
5- TNF-α + LAD;
6- TNF-α + LAD + INS.

FIGURE 8

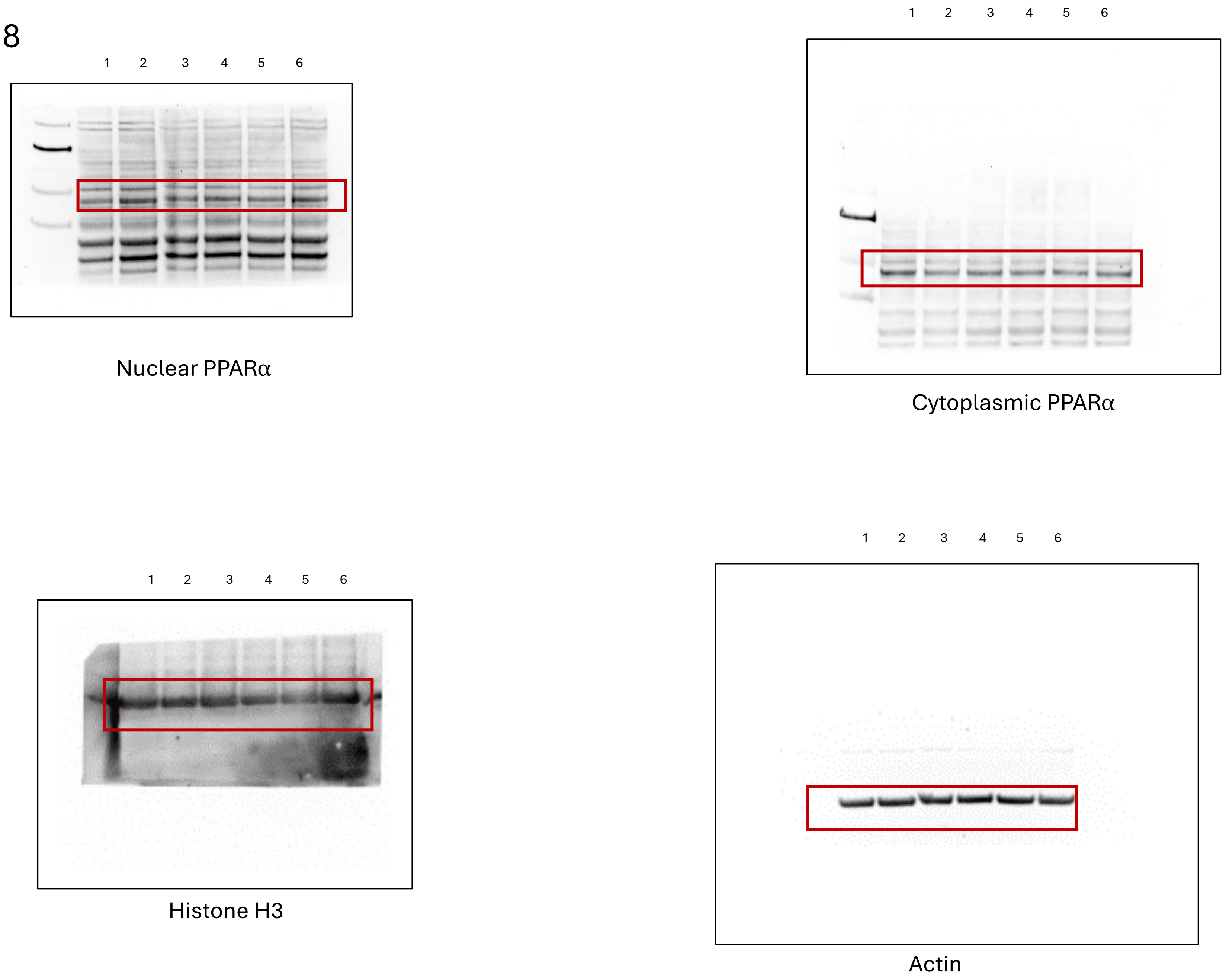

Legend:

1-CTR;
2-CTR + INS;
3- TNF-α;
4- TNF-α + INS;
5- TNF-α + LAD;
6- TNF-α + LAD + INS.
